# Comprehensive Analysis of the Fourteen Complete Genome Sequences of *Buchnera aphidicola* Isolated from *Aphis* Species

**DOI:** 10.4014/jmb.2409.09004

**Published:** 2024-12-19

**Authors:** Jin-Ho Yun, Jongsun Park, Hong Xi, Sangjune Nam, Wonhoon Lee, Seong-Ki Kim

**Affiliations:** 1 Cell Factory Research Center, Korea Research Institute of Bioscience and Biotechnology KRIBB), Daejeon 34141, Republic of Korea; 2 Department of Environmental Biotechnology, KRIBB School of Biotechnology, University of Science & Technology, Daejeon 34113, Republic of Korea; 3 Department of Integrative Biotechnology, Sungkyunkwan University, Suwon 16419, Republic of Korea; 4 Infoboss Inc., Seoul 06088, Republic of Korea; 5 Infoboss Research Center, Seoul 06088, Republic of Korea; 6 Agricultral Corporation Jeju Chunji, Jeju 63036, Repulic of Korea; 7 Department of Plant Medicine and Institute of Agriculture & Life Science, Gyeongsang National University, Jinju 52828, Republic of Korea; 8 Department of Life science, Chung-Ang University, Seoul 06974, Republic of Korea

**Keywords:** *Buchnera aphidicola*, endosymbiont bacterial genome, *Aphis*, nucleotide diversity, intraspecific variations, simple sequence repeats, phylogenetic analysis

## Abstract

Endosymbionts are important for insect species as they provide essential substances to the host. Due to the technical advance of NGS technology and *de novo* assemblers, many endosymbionts bacterial genomes are available now. Here, we analysed fourteen endosymbiont bacterial genomes of *Aphis* genius, one of notorious pest species. Fourteen genomes displayed the length between 628,098 bp to 634,931 bp; GC ratio was from 24.2 % to 25.6 % with no structural variation found. The nucleotide diversity distribution across the 14 endosymbiont genomes revealed three distinct regions, each separated by varying levels of nucleotide diversity. Intraspecific variations identified from endosymbiont bacterial genomes of the same host species revealed numbers of SNPs ranging from 31 (0.0049%) to 1,652 (0.26%) and those of INDELs ranging from 7 (21 bp; 0.0033%) to 104 (285 bp; 0.0045%). 250 unique SSRs, 28 different common SSR groups, and one different SSR group in two genomes were identified and used as a potential molecular marker to distinguish intraspecific population. Phylogenetic analysis further showed congruence between the endosymbiont bacterial genomes and the host species phylogeny, except *Aphis nasturtii*, *Aphis helianth*, and *Aphis auranti*, which require additional endosymbiont genomes for clarification. This comparative analysis result could serve as a cornerstone for understanding the relationship between host and endosymbiont species from a genomic perspective.

## Introduction

Endosymbionts are important for insect species as they can help digestion of host species by providing essential substances [1, 2]. In particular, most of aphids contain endosymbiotic bacteria identified as *Buchnera aphidicola* [3, 4], which provides essential amino acids and other substances unable to be synthesized by the host [5]. Conversely, aphids, host species, developed bacteriocyte cells, specialized in maintaining tight symbiotic relationship with *B. aphidicola* [6].

Due to the development of next generation sequencing (NGS) technologies, endosymbiont genomes can be easily assembled from the samples originated from the host species [7-10]. With this advantage, at least one hundred complete *Buchnera* genomes have been sequenced [5, 11-18], of which length ranges from 395,344 (CP135012) to 671,355 bp (CP084934), that is between those of ant endosymbiont *Blochmannia* (705,557 to 791,645 bp) [19-25] and Cicadidae endosymbiont (*Candidatus Hodgkinia cicadicola*; 96,363 to 150,297 bp) [26, 27].

The *Aphis* genus, part of the Aphididae family, contains many notorious agricultural pests including *Aphis glycine* and *Aphis gosypii* [28]. Previously, several genomic studies, including the sequencing of whole genomes [29-31] and mitochondrial genomes [32-38] have been conducted. As of now, fourteen complete genome sequences of symbiont of *Aphis* genus have been sequenced and are available in NCBI (Table 1). Notably, phylogenetic relationship of the host species displayed a very similar trend with that of endosymbiont bacterial genomes, but only two species of *Aphis* genus were included [39], lacking enough information to elucidate detailed relationship between host and endosymbionts in *Aphis* genus.

In this study, we conducted a comparative analysis of fourteen endosymbiont genomes from *Aphis* genus. These genomes range from 628,098 bp to 634,931 bp, with GC ratio from 24.2% to 25.6%. By examining nucleotide diversity, intraspecific variations, and the presence of simple sequence repeats (SSRs), this study aims to uncover the genetic diversity of these endosymbionts and explore their potential use as molecular markers for distinguishing different populations. Additionally, the study seeks to compare the phylogenetic relationships between the host species and their endosymbionts, with a focus on highlighting congruences or discrepancies that may provide new insights into the co-evolutionary dynamics between *Aphis* species and their symbionts.

## Materials and Methods

### Adjustment of Circular Bacterial Genome Sequences

Due to the usual circular form of bacterial genomes, , there was no standard position as the starting point of the genome. Among 14 endosymbiotic bacterial genomes identified from *Aphis* genus, four genomes (*i.e.*, CP042427, CP042426, CP056772, and CP056771) were adjusted based on multiple-time pair-wise alignments with the remaining genomes with MAFFT 7.450 [40] because the starting position of these genomes are different from the remaining genomes. The specific regions located at the end of sequences displayed via pair-wise alignment were moved to the end of genome sequences under the environment of Geneious Prime 2024.0.7 (Biomatters Ltd., New Zealand). After rearrangement, pair-wise alignment was conducted again to confirm whether the genome sequences were suitably rearranged.

### Nucleotide Diversity Analysis

Nucleotide diversity was calculated using the method proposed by Nei and Li [41] based on the multiple sequence alignments of the fourteen endosymbiont genomes using the module of Genome Information System (GeIS; https://geis.infoboss.co.kr) which have been adapted in various genomic studies [42-44]. The window size was set at 500 bp and the step size was 200 bp when using the sliding-window method, the conditions of which were similar with the previous studies [45-47].

### Identification of Intraspecific Variations

Single nucleotide polymorphisms (SNPs) and insertions and deletions (INDELs) were identified from the pair-wise sequence alignments with eight endosymbiont genomes of *A. rurantii*, *A. craccivora*, and *A. gossypii* using MAFFT 7.450 (Katoh and Standley 2013) with ‘Find variations/SNPs’ implemented in Geneious Prime 2024.0.7 (Biomatters Ltd.), based on previous studies [48-50]. Distributions of SNPs and INDELs were calculated by the GeIS modules with the INDEL region defined as continuous INDELs.

### Identification and Comparison of Simple Sequence Repeats (SSRs)

Simple Sequence Repeats (SSRs) were identified based on the endosymbiotic bacterial genome sequence using the pipeline of the SSR database (SSRDB; http://ssrdb.infoboss.co.kr/), based on previous genomic studies [10, 51-53]. Being recognized as the nucleotide array composed of repeats with one or up to six base pair units, monoSSR indicated an array of nucleotide repeats containing a particular base and hexaSSR denoted an array of nucleotide repeats containing six base pair unit. The overall length of SSR was mostly over 10 base pairs. Moreover, the classification of SSR was performed with additional criteria based on previous analyses to facilitate better understanding of SSR patterns [54-62].: (1) conventionally-defined ‘normal SSR’ from monoSSR to hexaSSR (2) ‘extended SSR’ referring to SSRs with repeats from 7 bp unit (heptaSSR) to 10 bp unit (decaSSR) (3) ‘potential SSR’ referring to specific cases with only two units in pentaSSR and hexaSSR, while three or more units of pentaSSR and hexaSSR were classified as ‘normal SSR.’

### Comparison of Simple Sequence Repeats among Fourteen Endosymbiont Bacterial Genomes

The SSR database provided a comparison pipeline based on the flanking sequences of identified SSRs (*i.e.*, SSR Comparison History). While nucleotide coordination was not absolute criterion to identify common SSRs because of different coordination of the endosymbiont bacterial genomes, the pipeline identified similar SSRs by selecting SSRs with similar 60-bp flanking sequences with both sequence coverage and identity above 90% on both sides. After selecting the candidates of similar SSRs, unit sequence and repeat number of each SSR were investigated to understand whether sets of similar SSRs, namely group SSRs, contained similar SSR or not [52, 63-65].

### Construction of Phylogenetic Trees

Fourteen complete endosymbiont genomes with one outgroup endosymbiont genome of *Buchnera aphidicola* (*i.e.*, an endosymbiont of *Hyperomyzus lactucae*; CP034876), were aligned by MAFFT 7.450 [40] and the quality of multiple sequence alignments was examined manually. In addition, to understand phylogenetic relationship between the host species, their complete mitochondrial genomes were considered. Although *A. helianthin*, *A. nasturtii*, *A. urticata* did not have any complete mitochondrial genomes, *COI* sequences originated from the host *Aphis* species and *Hyperomyzus lactucae* were instead obtained from NCBI. For the endosymbiont bacterial genomes, the neighbor-joining (NJ) and Bayesian inference (BI) trees were reconstructed using Geneious Tree Builder (Geneious Prime 2024.0.7) and MrBayes 3.2.6 [66], respectively. During the NJ analysis, Tamura-Nei model was deployed as a genetic distance model with random seeds of 468,795 and a support threshold of 50%. Bootstrap analyses with 5,000 pseudoreplicates were conducted. For BI analysis, the HKY85 model with gamma rates was used as a molecular model; Markov Chain Monte Carlo (MCMC) algorithm was also employed for 1,100,000 generations along with sampling trees for every 200 generations with four chains running simultaneously. Trees from the first 100,000 generations were discarded as burn-in. For the *COI* sequences of host species, sequence alignment using MAFFT 7.450 [40] was performed under identical conditions; Maximum Likelihood (ML) tree was constructed with 5,000 bootstrap repeats using MEGA 11.0.13 [67].

## Results and Discussion

### Comparison of Fourteen Complete *Buchnera aphidicola* Genomes from *Aphis* Species

**Fig. 1:**
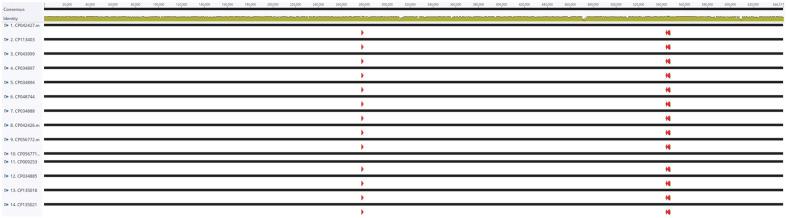
Multiple sequence alignment of fourteen endosymbiont bacterial genomes of *Aphis* genus. Black bar on the top represents endosymbiont bacterial genome with genomic coordination. Below the black bar, dark-yellow graph presented the nucleotide identity in each coordination. Accession of endosymbiont bacterial genomes was displayed in the left part. Red arrows indicate ribosomal RNAs.

Fourteen complete genomes of *Buchnera aphidicola* isolated from *Aphis* genus aphids were downloaded from the NCBI (Table 1) and prepared for further comparisons (See Materials and Methods). Multiple sequence alignments of the fourteen genomes indicated that there was no structural variation among them (Fig. 1), suggesting a well-conserved genomic structure, consistent with previous studies on complete genomes of other endosymbionts, including *Candidatus Riesia pediculicola* [68].

**Fig. 2:**
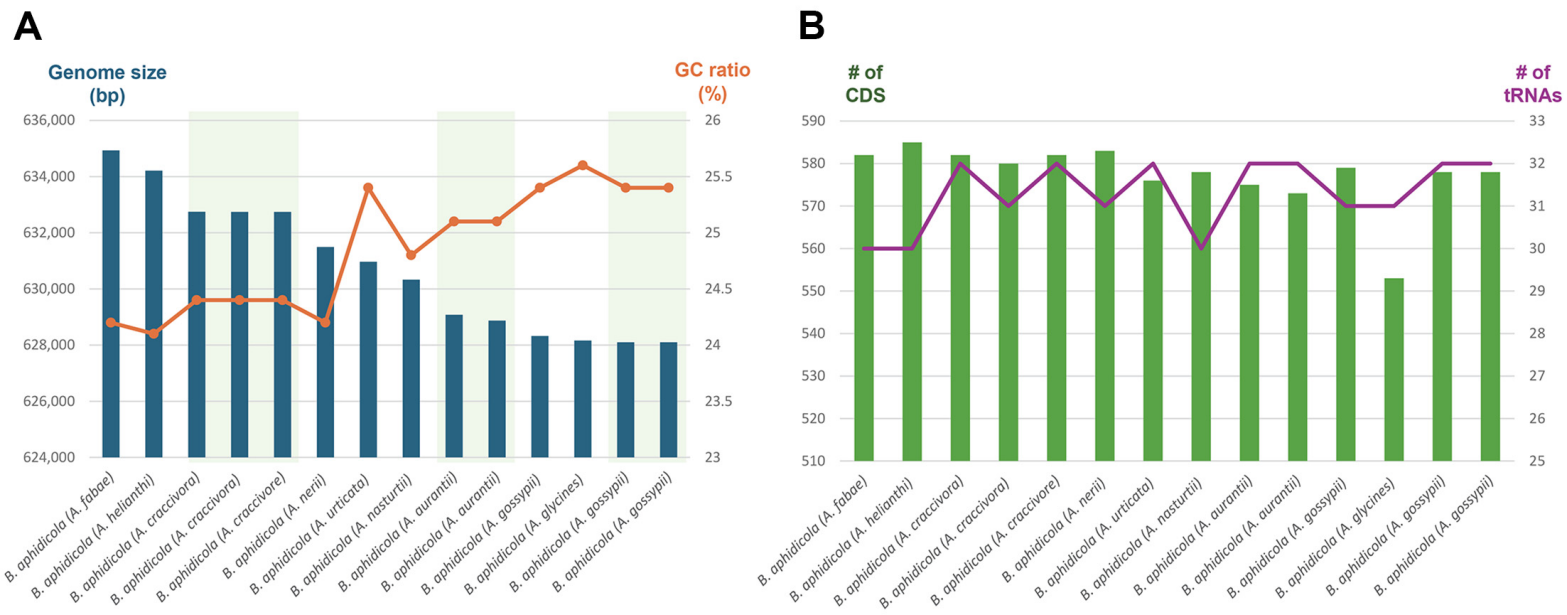
Genome size, GC ratio, number of CDs and tRNAs of the fourteen endosymbiont bacterial genomes. (A) X-axis means fourteen endosymbiont bacterial genomes and Y-axis indicates genome size and GC ratios presented by blue bars and yellow line, respectively. (**B**) X-axis means fourteen endosymbiont bacterial genomes and Y-axis indicates the number of CDs exhibited as green bars and number of tRNAs displayed as purple line.

The genome length of the fourteen *B. aphidicola* genomes ranged from 628,098 bp to 634,931 bp, and their GC content ranged from 24.2 % to 25.6 % (Fig. 2A). Interestingly, the GC content of endosymbionts originating from the same *Aphis* species was identical (Fig. 2A), indicating host-dependent similarity in endosymbiont bacterial genomes. In contrast, the numbers of coding sequences (CDSs; 553 to 585) and transfer RNAs (tRNAs; 30 to 32) in the fourteen bacterial genomes did not exhibit strong host specificity (Fig. 2B). In addition, the number of ribosomal RNAs (rRNAs) was identical across genomes at three, and the numbers of non-coding RNAs (ncRNAs) and tRNAs ranged from 1 to 2 and 0 to 1, respectively (Table 1).

### Identification of a Three-Region Structure in Fourteen Endosymbiont Genomes

**Fig. 3:**
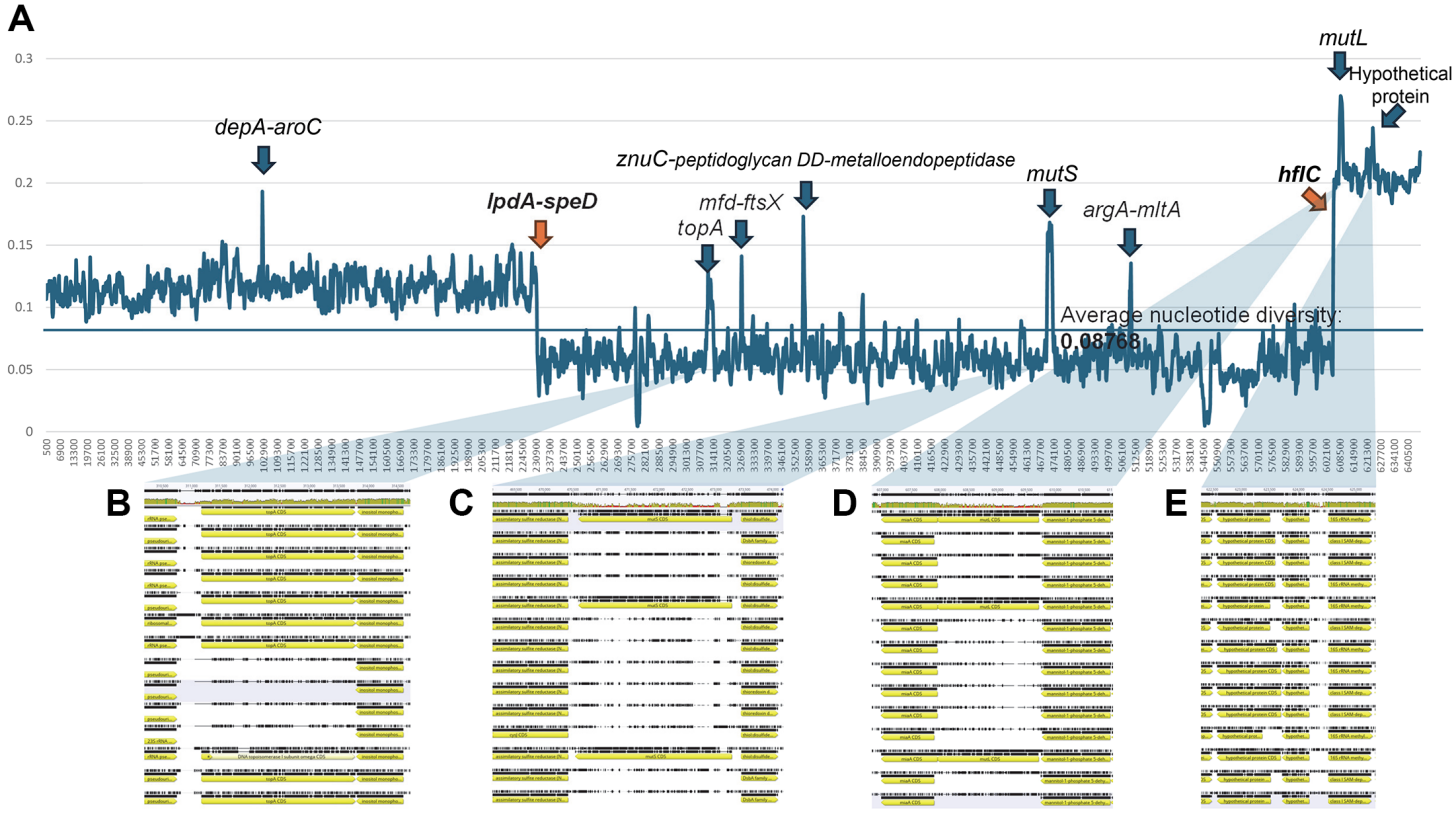
Nucleotide diversity of the fourteen endosymbiont bacterial genomes. (**A**) X-axis indicates genomic coordination of multiple sequence alignment of fourteen endosymbiont bacterial genomes and Y-axis presents nucleotide diversity. Orange allows indicate the border of different nucleotide diversity levels and blue arrows exhibit peaks of nucleotide diversity with genes around the position. (**B-E**) presented the multiple sequence alignments in specific genes containing nucleotide diversity peak.

**Fig. 4:**
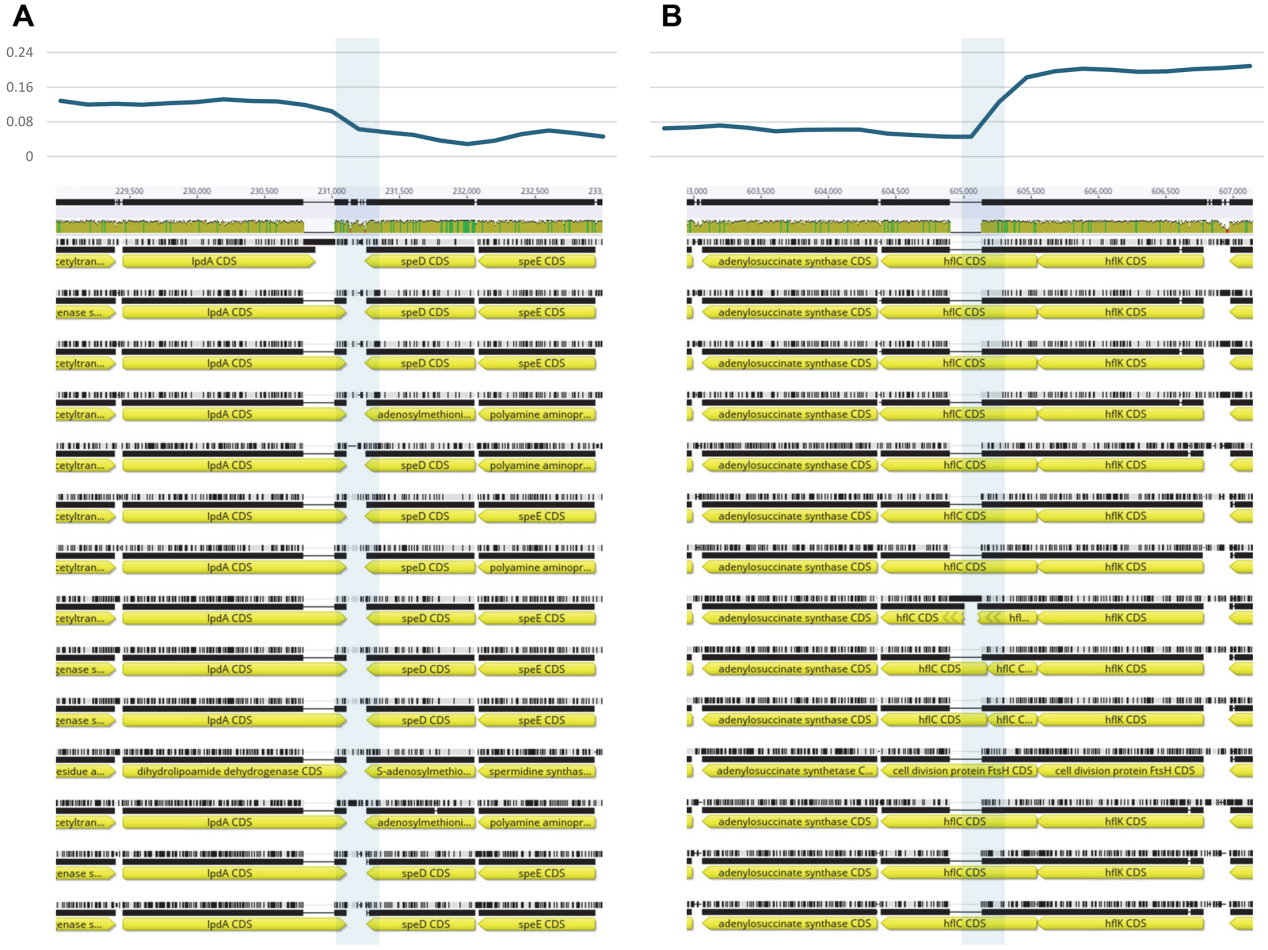
Nucleotide diversity distribution at border of different regions. X-axis indicates genomic coordinates and Yaxis indicates nucleotide diversity. Transparent light-blue boxes present the region where nucleotide diversity dramatically changed. Below graph, multiple sequence alignments were presented with yellow arrow as CDs.

To understand genome-wide variation in the fourteen endosymbiont genomes, nucleotide diversity was calculated from multiple sequence alignments (see Materials and Methods). Average nucleotide diversity was 0.08768, and three distinct regions were identified based on the nucleotide diversity trend calculated using the sliding window method (Fig. 3). Specifically, the first boundary between two of the identified regions was located between *ipdA* (lipoamide dehydrogenase) and *speD* (S-adenosylmethionine decarboxylase proenzyme; Fig. 4A). The second boundary, separating the next identified region, was found within *hflC* (an inner membrane protein that forms part of the HflCK complex, which interacts with and regulates the ATP-dependent protease *FtsH* in *Escherichia coli*; Fig. 4B).

This mosaic structure resembles that of the chloroplast genome, which comprises four different regions: the large single copy (LSC), small single copy (SSC), and two identical inverted repeats [69]. Chloroplast genes in the LSC region are involved in photosynthesis, metabolism, and other cellular processes, whereas those in the SSC region contribute to RNA processing and translation [70]. Although further studies are needed to elucidate the specific functions of each of these regions in endosymbionts, the three-region structure in the bacterial genomes may suggest that these regions have been subject to different selective pressures and were merge at a certain evolutionary time point.

### Identification of Highly Variable Regions in Fourteen Endosymbiont Bacterial Genomes

At least eight peaks of nucleotide diversity were identified (Fig. 3A): *depA*-*aroC*, *topA* (Type I DNA topoisomerase), *mfd*-*ftxS*, *znuC*-peptidoglycan DD-metalloendopeptidase, *mutS*, *argA*-*mltA*, *mutL*, and a hypothetical protein. Four of these peaks corresponded to typical intergenic regions, while three out of eight peaks (*topA*, *mutS*, *mutL*) suggested possible gene gain and/or gene loss (Fig. 3B-3D). In the case of *topA*, four out of the fourteen endosymbiotic bacterial genomes – three from *Aphis gossypii* and one from *Aphis glycines* – appeared to have lost *topA* (Fig. 3B). Additionally, the DNA mismatch repair proteins *MutS* and *MutL* were lost in 11 out of 14 endosymbiont genomes (Fig. 3C and 3D). All three genes are crucial for precise DNA replication. This phenomenon suggests that four endosymbionts have found a way to survive without the essential genes of DNA replication, reflecting that endosymbionts can lose many essential genes and still thrive [71]. The hypothetical protein and intergenic space between the hypothetical protein and 16S rRNA methyltransferase exhibited higher nucleotide diversity (Fig. 3E). This observation suggests that the loss of *topA* may have occurred before the divergence of the common ancestor of *A. gossypii* and *A. glycines*.

The results also indicated the gain of *mutS* and *mulT* genes. Specifically, the presence of *mutS* and *mulT* was observed in the endosymbiont of *A. fabae*, *A. helianthi*, and *A. nerii*, which occupy different phylogenetic positions [72]. This suggests that the acquisition of these genes may have occurred independently through convergent evolution. Lastly, the hypothetical protein, which displayed high nucleotide diversity (Fig. 3E), was conserved across the fourteen endosymbiont genomes, implying that its function may have been maintained throughout evolution. Notably, each of these identified genes may play a role in shaping the symbiotic relationships between the endosymbionts and their aphid hosts. Therefore, further systematic investigations into these identified genes and the observed genomic variations could also have practical implications, including potential applications in pest management.

### Investigation of Intraspecific Variation in Endosymbiont Bacterial Genomes

**Fig. 5:**
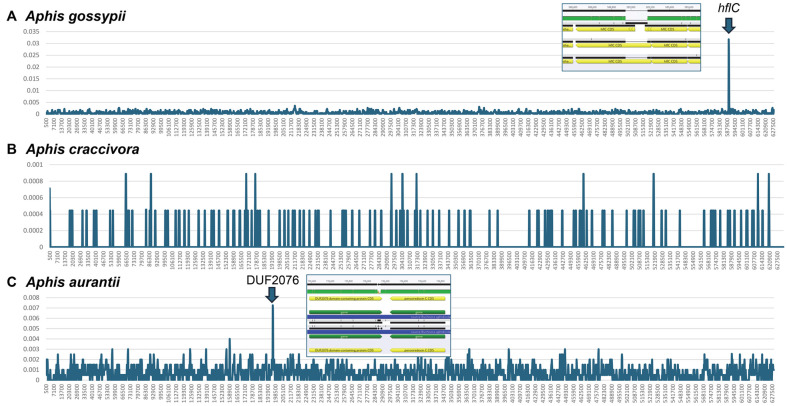
Nucleotide diversity of three multiple sequence alignments of endosymbiont bacterial genomes of three *Aphis* species. X-axis presented genomic coordination of multiple sequence alignment and y-axis presented nucleotide diversity. Blow arrows mean peaks of nucleotide diversity and multiple sequence alignment results around peak were presented in the blue-bordered boxes.

Three host species – *A. gossypii*, *A. craccivora*, and *A, aurantii* – contained more than two endosymbiont bacterial genomes; thus, they served as suitable organisms to understand intraspecific variations within endosymbiont bacterial genomes. The average nucleotide diversity of *A. gossypii* and *A. aurantii* was 0.0005941 and 0.0006659, respectively, while that of *A. craccivora* was 0.00005024. The endosymbiotic bacterial genomes of *A. craccivora* exhibited almost flat nucleotide diversity (Fig. 5B), while each of *A. gossypii* and *A. aurantia* presented one peak (Fig. 5A and 5C). The highest peak in *A. gossypii* (Fig. 5A) was formed by gap sequences in *B. aphidicola* (*i.e.*, an endosymbiont of *A. gossypii*) strain Ago-UT1 (CP042426). The 3’ region of DUF2076, a putative periplasmic ligand-binding sensor protein, resulted in the highest nucleotide diversity peak (Fig. 5C). These results indicate that the nucleotide diversity of endosymbionts within the same host species was extremely low, even though two peaks were identified in the multiple sequence alignments due to structural variations in the specific regions.

**Fig. 6:**
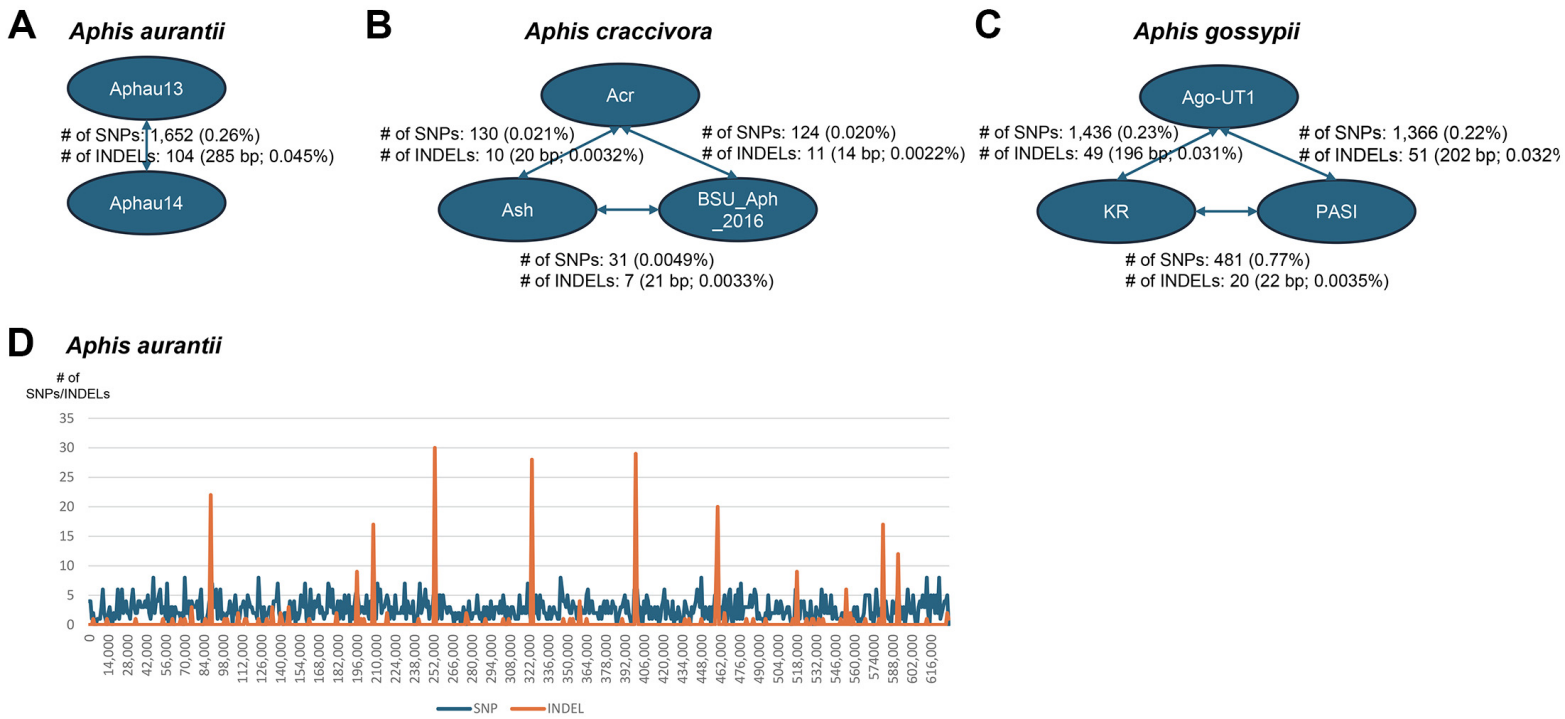
Intraspecific variations identified from three multiple sequence alignments of endosymbiont bacterial genomes of three *Aphis* species. (**A-C**) Presents intraspecific variations of pair-wise comparison of endosymbiont bacterial genomes. Blue round boxes indicate endosymbiont bacterial genomes and arrows mean pair-wise comparison with number of SNPs and INDELs. (**D**) Exhibits the distribution of intraspecific variations along with bacterial genome. X-axis genomic coordination of sequence alignment and Y-axis indicate number of SNPs (blue line) and INDELs (yellow line).

The pair-wise alignments of the eight endosymbiont bacterial genomes also indicated intraspecific variations. In particular, the number of SNPs ranged from 31 (0.0049%) to 1,652 (0.26%), and the number of INDELs ranged from 7 (21 bp; 0.0033%) to 104 (285 bp; 0.0045%) (Fig. 6). Notably, the proportion of SNPs and INDELs were lower than those in the mitochondrial genomes of the corresponding hosts. For instance, the proportion of SNPs found in the mitochondrial genomes of *Aphis gossypii* was around 0.55% (88 SNPs from 16,044 bp mitochondrial genome) [37]. While the distribution of SNPs and INDELs from the seven pair-wise comparisons was similar to that of nucleotide diversities, showing a periodical repetition of high-density INDELs in the endosymbiont bacterial genomes of *Aphis aurantia* (Fig. 6D), these results could offer important clues to understanding how these genomes have adapted within their host species.

### Comparative Analysis of Simple Sequence Repeats in Fourteen Endosymbiont Bacterial Genomes

**Fig. 7:**
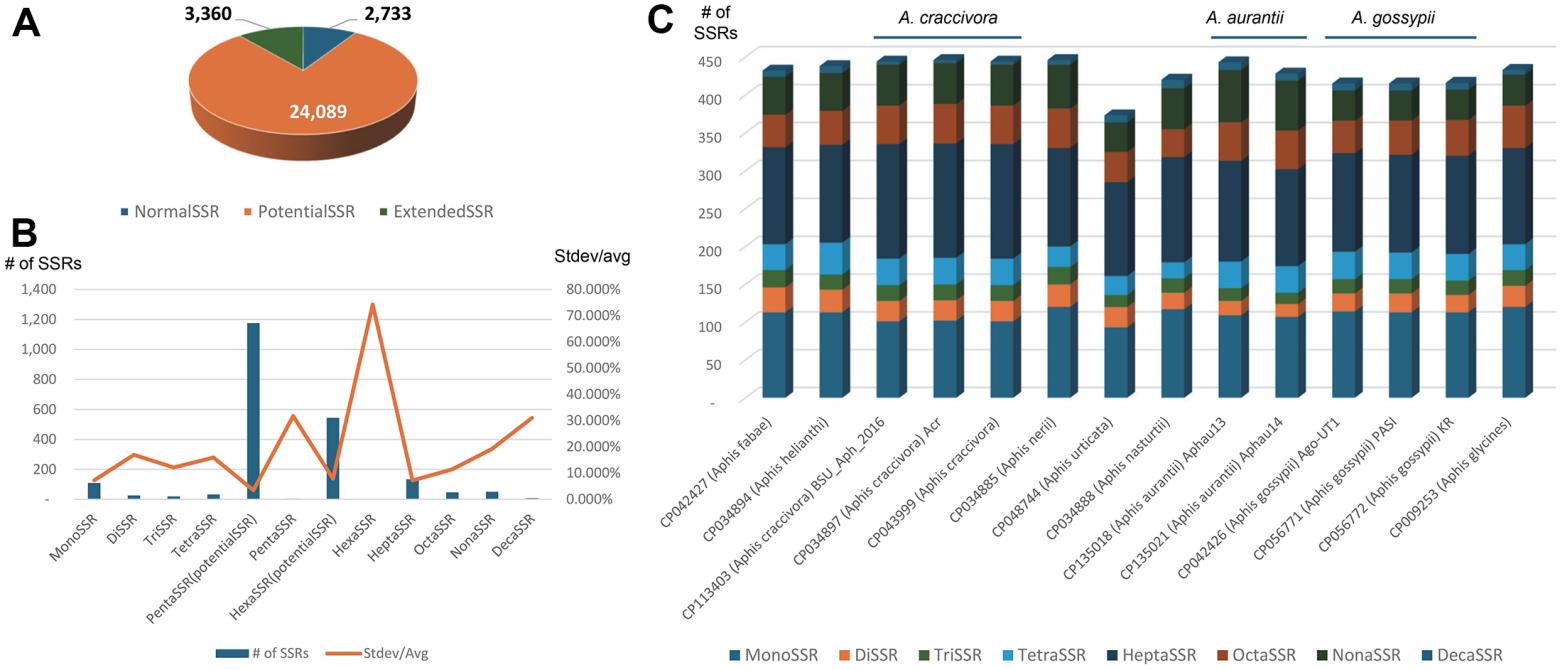
Simple sequence repeats identified from fourteen endosymbiont bacterial g genomes of *Aphis* genus. (**A**) Pie graph displays number of normal SSRs (blue), potential SSRs (orange), and extended SSRs (green) from 14 endosymbiont bacterial genomes. (**B**) X-axis indicates types of SSRs and Y-axis displays number of SSRs (blue bar) and ratio between standard deviation and average number of SSRs (orange line). (**C**) X-axis indicates 14 endosymbiont bacterial genomes and Y-axis indicates number of SSRs. Blue lines upper the graph mean the host species of endosymbiont bacterial genomes is same.

Simple sequence repeats (SSRs) refer to short nucleotide sequences repeated multiple times in tandem within a sequence [73]. SSRs have been utilized as useful molecular markers to distinguish species [74, 75] and to isolate targeted bacterial artificial genomes (BACs) [76, 77]. Analysis of fourteen bacterial genomes identified 2,733 normal SSRs, 24,089 potential SSRs, and 3,360 extended SSRs (Fig. 7A). The total number of SSRs ranged from 2,029 (CP048744) to 2,303 (CP042427) with a standard deviation of 89.08 and a coefficient of variation of 4.132%. Variations across different SSR types (*i.e.*, 6 normal SSRs, 2 potential SSRs, and 4 extended SSRs) were substantial, showing the lowest coefficient of variation in potential pentaSSRs (3.45%) and the highest in hextSSRs (74.28%; Fig. 7B). Among the ten types of normal and extended SSRs, monoSSRs and heptaSSRs exhibited coefficients of variation of 7.26% and 7.19%, respectively (Fig. 7B).

The endosymbiont bacterial genome of *A. urticata*, displayed the lowest number of SSRs, containing only monoSSRs, triSSRs, octaSSRs, and nonaSSRs (Fig. 7C). Three host species, *A. craccivora*, *A. gossypii*, and *A. aurantia*, each with more than one endosymbiont bacterial genome, exhibited low variation in SSR numbers across each SSR type, except *A. aurantia* (Fig. 7C).

**Fig. 8:**
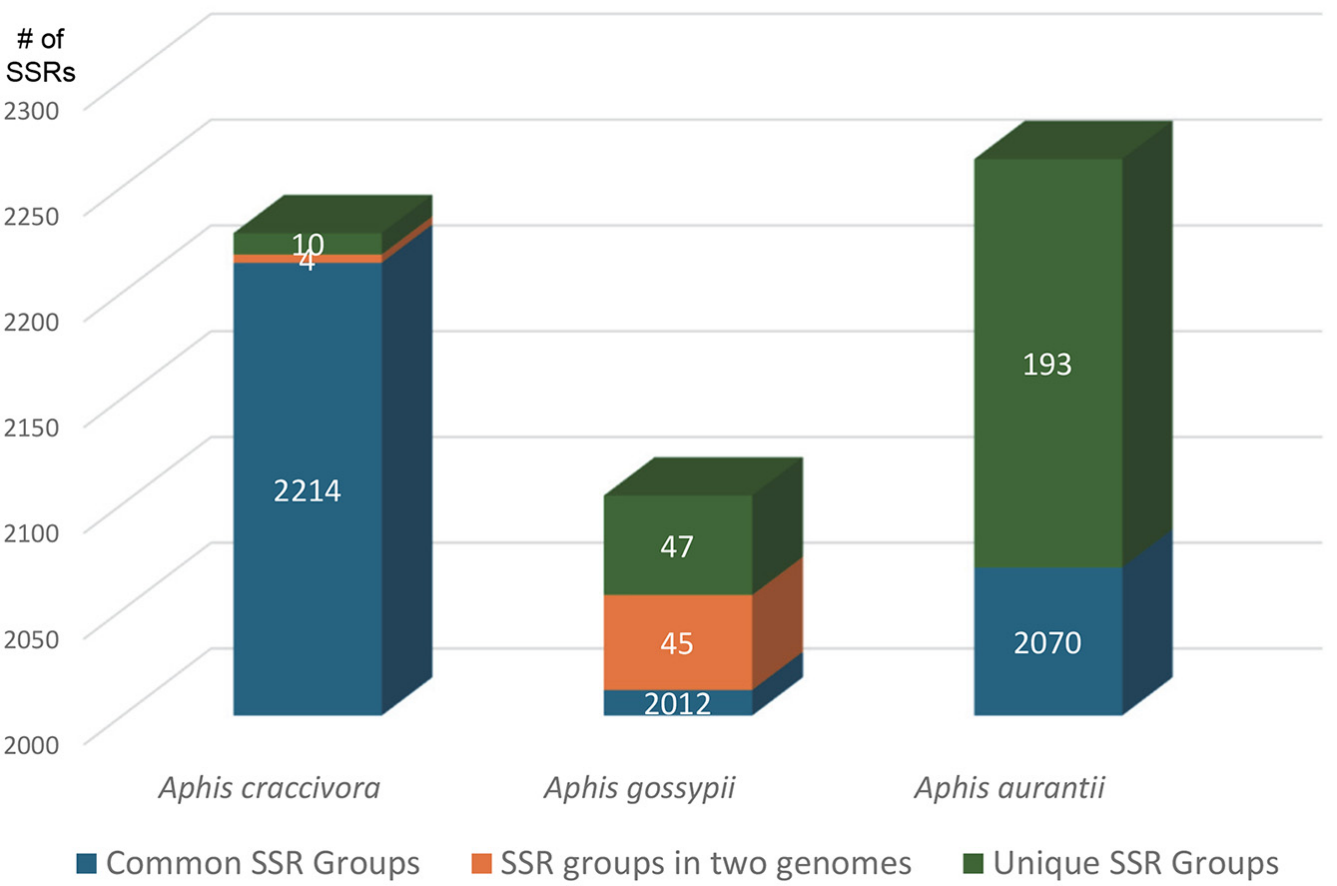
SSR groups identified from endosymbiont bacterial genomes of three *Aphis* species. X-axis indicates three Aphis host species and Y-axis means number of SSRs starting with 2,000.

To understand SSR differences within the same host species, SSRs identified from the endosymbiont bacterial genomes of three *Aphis* species (*i.e.*, *A. craccivora*, *A. aurantia*, and *A. gossypii*) were compared. Among the three endosymbiont genomes of *A. craccivora*, only 10 unique SSRs (0.45%) were found in one genome, while 4 SSR groups were found in two genomes (0.18%; Fig. 8A). In contrast, *A. gossypii* endosymbiont genomes contained 47 unique SSRs (2.23%) and 45 SSR groups shared between two genomes (2.14%; Fig. 8). The two endosymbiont bacterial genomes of *A. aurantii* displayed 183 unique SSRs (8.53%; Fig. 8). Additionally, variation in repeat numbers was observed in common SSR groups: 9 out of 2,205 in *A. craccivora*, 14 out of 1,998 in *A. gossypii*, and 5 out of 2,065 in *A. aurantii*. In *A. craccivora*, one SSR was unique to two endosymbiont genomes, indicating that these unique SSRs could serve as valuable markers for distinguishing intraspecific populations.

### Phylogenetic Analysis of Fourteen Endosymbiont Bacterial Genomes

**Fig. 9:**
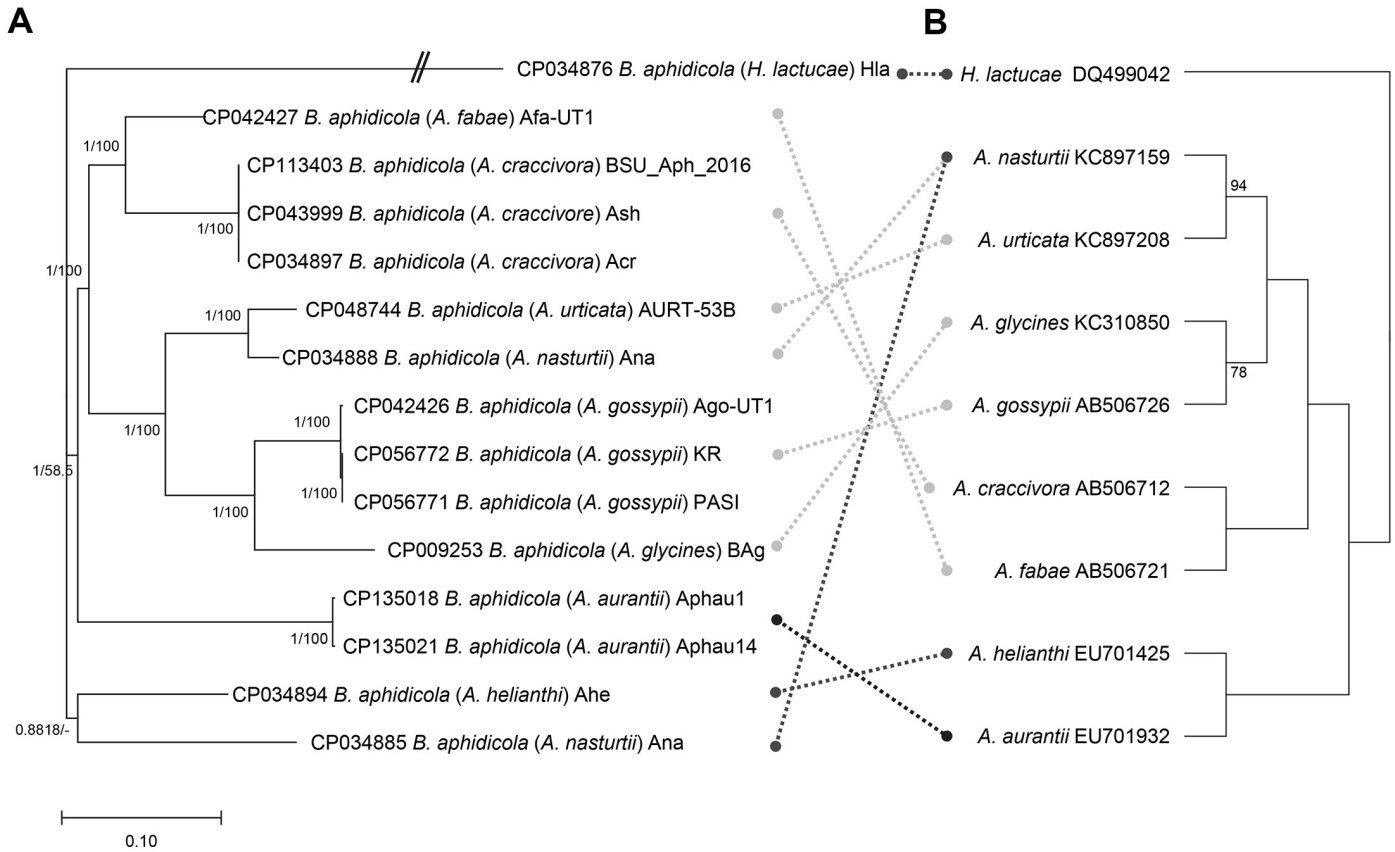
Phylogenetic tree of endosymbiont bacterial genomes with host phylogeny. (**A**) display phylogenetic tree of fifteen endosymbiont bacterial genomes including outgroup species. Phylogenetic tree was drawn based on BI tree. The numbers above the branches are posterior probabilities from the BI tree and the bootstrap support values of the NJ. (**B**) phylogenetic tree was drawn based on ML tree. The number above the branches is the bootstrap support values of the ML. Grey dotted lines indicate the same phylogenetic position between endosymbiont bacterial genome and host species and black dotted lines means incongruent cases.

It is worth nothing that the evolutionary history of endosymbionts is strongly dependent on that of their corresponding host species. Based on the alignment of fourteen complete endosymbiont bacterial genomes, two out of the three *Aphis* species containing more than one endosymbiont genome clustered together with high support values, while *A. nasturtii* clustered separately, along with *A. helianthi* and *A. urticata*, respectively (Fig. 9A). Possible reasons for the separate clustering of the two endosymbiont genomes of A. nasturti include: (1) the presence of several cryptic species in A. nasturti as reported in *A. gossypii* [78]; (2) horizontal gene transfer between endosymbiont bacteria, potentially between *A. nasturtii* and *A. helianthin*, which share some overlapping host plant species (*e.g.*, sunflower) [69]; and (3) misidentification of the host species associated with these endosymbiont genomes. This inconsistency may be resolved as additional endosymbiont bacterial genomes become available.

To explore the evolutionary relationship between host and endosymbiont bacterial species, a phylogenetic tree of the host species was further constructed (Fig. 9B; See Material and Methods). Due to the limited length of cytochrome oxidase I sequences available across the host species [23], the support values for the phylogenetic tree were not substantially high; however, the overall topology was consistent with previous phylogenetic studies [72, 79]. Inconsistencies in the host species phylogenetic tree were noted in the endosymbiont genomes of *A. aurantia* and *A. helianthin*, influenced by one of the endosymbiont genomes of *A. nasturtii* (CP034885; Fig. 8). Given the notable intraspecific variations between the two endosymbiont genomes of *A. nasturtii* (Fig. 6A), additional endosymbiont genomes of *A. asturtii*, *A. aurantia*, and *A. helianthin* are needed to clarify the phylogenetic relationships among these endosymbiont bacterial species.

## Figures and Tables

**Table 1:** List of available *Buchnera aphidicola* genomes isolated from *Aphis* genus.

No	Species	Strain	GenBank	Length (bp)	GC (%)	# of PCGs	# of tRNAs	# of rRNAs	# of ncRNA	# of tmRNAs	Reference
1	*Buchnera aphidicola* (*A. fabae*)	Afa-UT1	CP042427	634,931	24.2	582	30	3	2	1	[80]
2	*Buchnera aphidicola* (*A. helianthi*)	Ahe	CP034894	634,211	24.1	585	30	3	2	1	[81]
3	*Buchnera aphidicola* (*A. craccivora*)	BSU_Aph_2016	CP113403	632,746	24.4	582	32	3	1	1	N/A
4	*Buchnera aphidicola* (*A. craccivora*)	Acr	CP034897	632,742	24.4	580	31	3	1	1	[81]
5	*Buchnera aphidicola* (*A. craccivore*)	Ash	CP043999	632,741	24.4	582	32	3	1	1	N/A
6	*Buchnera aphidicola* (*A. nerii*)	Ane	CP034885	631,491	24.2	583	31	3	2	1	[81]
7	*Buchnera aphidicola* (*A. urticata*)	AURT-53B	CP048744	630,969	25.4	576	32	3	2	1	[82]
8	*Buchnera aphidicola* (*A. nasturtii*)	Ana	CP034888	630,331	24.8	578	30	3	2	1	[81]
9	*Buchnera aphidicola* (*A. aurantii*)	Aphau13	CP135018	629,080	25.1	575	32	3	2	1	[83]
10	*Buchnera aphidicola* (*A. aurantii*)	Aphau14	CP135021	628,870	25.1	573	32	3	2	1	[83]
11	*Buchnera aphidicola* (*A. gossypii*)	Ago-UT1	CP042426	628,324	25.4	579	31	3	2	1	[80]
12	*Buchnera aphidicola* (*A. gossypii*)	KR	CP056772	628,100	25.4	578	32	3	2	1	N/A
13	*Buchnera aphidicola* (*A. gossypii*)	PASI	CP056771	628,098	25.4	578	32	3	2	1	N/A
14	*Buchnera aphidicola* (*A. glycines*)	BAg	CP009253	628,164	25.6	553	31	3	1	0	[84]
